# Sestrin2 inhibits mTORC1 through modulation of GATOR complexes

**DOI:** 10.1038/srep09502

**Published:** 2015-03-30

**Authors:** Jeong Sig Kim, Seung-Hyun Ro, Myungjin Kim, Hwan-Woo Park, Ian A. Semple, Haeli Park, Uhn-Soo Cho, Wei Wang, Kun-Liang Guan, Michael Karin, Jun Hee Lee

**Affiliations:** 1 Department of Molecular & Integrative Physiology, University of Michigan, Ann Arbor, MI 48109, USA; 2 Department of Obstetrics and Gynecology, Soonchunhyang University Seoul Hospital, Seoul 140-743, Republic of Korea; 3 Department of Biological Chemistry, University of Michigan, Ann Arbor, MI 48109, USA; 4 Department of Pharmacology, University of California, San Diego, CA 92093, USA

## Abstract

Sestrins are stress-inducible metabolic regulators that suppress a wide range of age- and obesity-associated pathologies, many of which are due to mTORC1 overactivation. Upon various stresses, the Sestrins inhibit mTORC1 activity through an indirect mechanism that is still unclear. GATORs are recently identified protein complexes that regulate the activity of RagB, a small GTPase essential for mTORC1 activation. GATOR1 is a GTPase activating protein (GAP) for RagB whereas GATOR2 functions as an inhibitor of GATOR1. However, how the GATORs are physiologically regulated is unknown. Here we show that Sestrin2 binds to GATOR2, and liberates GATOR1 from GATOR2-mediated inhibition. Released GATOR1 subsequently binds to and inactivates RagB, ultimately resulting in mTORC1 suppression. Consistent with this biochemical mechanism, genetic ablation of GATOR1 nullifies the mTORC1-inhibiting effect of Sestrin2 in both cell culture and *Drosophila* models. Collectively, we elucidate a new signaling cascade composed of Sestrin2-GATOR2-GATOR1-RagB that mediates stress-dependent suppression of mTORC1 activity.

mTORC1 is a nutrient-sensing metabolic regulator that promotes protein and lipid anabolism and inhibits autophagic catabolism of nutrient deposits, protein aggregates and damaged organelles such as dysfunctional mitochondria^1^,^2^. Chronic activation of mTORC1 by overnutrition can result in diverse metabolic pathologies associated with aging, obesity and autophagic defects^1^. Upon chronic activation of mTORC1 as well as upon diverse environmental stresses, a family of stress-inducible proteins named Sestrins are induced through several stress-responsive transcription factors, such as p53, HIF-1, FoxO and c/EBPβ, and subsequently suppress mTORC1 activation^3^. In model animals such as *Drosophila* and mice, Sestrins are shown to be essential for maintaining metabolic homeostasis and preventing age- and obesity-associated pathologies^4^,^5^,^6^. Many of these pathologies are also suppressed by pharmacological or genetic inhibition of mTORC1/dTORC1^4^,^5^,^6^, suggesting that its ability to suppress mTORC1/dTORC1 activation is central for the Sestrins' metabolism-regulating role.

One possible mechanism of Sestrins-dependent mTORC1 regulation involves AMP-activated protein kinase (AMPK)^7^, which phosphorylates tuberous sclerosis complex 2 (TSC2) and Raptor and thereby inhibits mTORC1 activity^8^. It has been suggested that Sestrin2, the most thoroughly studied Sestrin isoform, associates with AMPK and promotes its activating phosphorylation by the upstream kinase LKB1^9^. Chemical or shRNA-mediated inactivation of AMPK prevented Sestrin2 from inhibiting mTORC1^7^ although the extent of the effect was varied depending on the types of cells and tissues. For example, Sestrin2 was still able to inhibit mTORC1 in HeLa cells, which do not express LKB1 and therefore exhibit very low amount of AMPK activity^3^. Therefore, it has been postulated that there could be additional mediators of Sestrin2 that suppress mTORC1 activation.

GATOR is a multiprotein complex that is composed of two subcomplexes called GATOR1 and GATOR2^10^. GATOR1 is composed of three proteins, DEPDC5, NPRL2 and NPRL3, whereas GATOR2 possesses five protein components, MIOS, WDR24, WDR59, SEH1L and SEC13^10^. GATOR1 serves as a GAP for RagB and its close homolog RagA, which are functionally redundant GTPases essential for mTORC1 activation during amino acid-rich conditions^11^,^12^,^13^, while GATOR2 inhibits the GAP activity of GATOR1^10^. GATOR1 is considered as a tumor suppressor as its absence can lead to constitutive activation of RagB and subsequent elevation of mTORC1 activity. Indeed, many human cancer cell lines have a deficiency in at least one of the three GATOR1 components, and loss of *DEPDC5* and *NPRL2* genes was observed in human glioblastoma and ovarian cancer tissues^10^. The mTORC1-regulating role of GATOR seems to be conserved in *Drosophila*^10^,^14^ and yeasts^15^,^16^,^17^. Although these findings implicate that GATOR1 and GATOR2 are important for mTORC1 regulation and tumorigenesis, it is not known how GATOR complexes themselves are physiologically regulated.

Here we show that Sestrin2 is a physiological regulator of GATOR complexes. By physically interacting with GATOR2, Sestrin2 releases GATOR1 from GATOR2-mediated inhibition. GATOR1 then inhibits RagB GTPase and subsequently prevents mTORC1 activation by amino acids. These results illustrate a new mechanism through which Sestrin2 negatively regulates mTORC1 and describe one way in which GATOR activity is regulated in mammalian and insect cells.

## Results

### Sestrin2 can regulate mTORC1 through an AMPK-independent pathway

**Figure 1:**
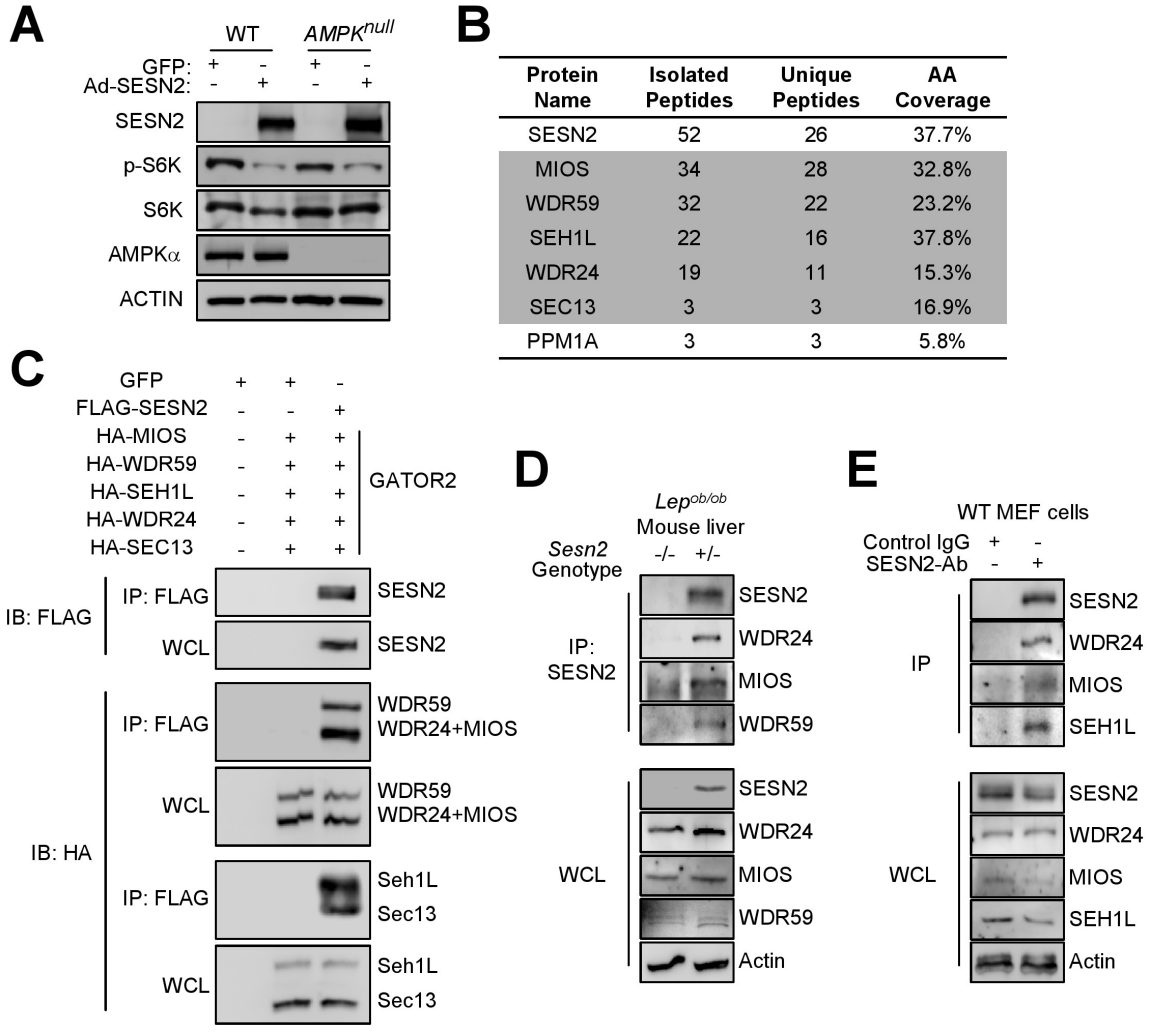
Identification of GATOR2 components as Sestrin2-binding partners. (A) Sestrin2 inhibits mTORC1 in *AMPK*-null cells. WT and *AMPK*-null (*Ampkα_1_^−/−^/Ampkα_2_^−/−^*) MEFs were infected with adenoviruses expressing GFP or Sestrin2 and analyzed through immunoblotting (IB). (B) Mass spectrometry analysis of Sestrin2-tandem affinity purification (TAP) products identified GATOR2 components as proteins that show the strongest physical interaction with Sestrin2. Sestrin2 TAP products from MCF10A cells were analyzed by MS-MS. The number of peptide hits for Sestrin2 and its six strongest interacting partners, among which five are GATOR2 components (shaded in grey), are shown. (C) GATOR2 as a complex shows strong physical association with Sestrin2 in HEK293 cells. Sestrin2 was co-transfected with all GATOR2 components as indicated. Sestrin2 was immunoprecipitated (IPed) with Flag antibody. Input (whole cell lysate, WCL) and IP complex were analyzed by IB. (D) Endogenous Sestrin2 interacts with endogenous GATOR2 proteins in livers from obese mice. Sestrin2 and its interacting proteins in liver lysates of 4-month-old *Lep^ob/ob^*/*Sesn2^+/−^* and *Lep^ob/ob^*/*Sesn2^−/−^* mice were IPed with Sestrin2 antibody. Input (WCL) and IP complex were analyzed by IB with indicated antibodies against endogenous proteins. (E) Endogenous Sestrin2 interacts with endogenous GATOR2 proteins in mouse embryonic fibroblast (MEF) cells treated with 100 μM etoposide, a DNA damage inducer that increases Sestrin2 expression, for 16 hrs. Sestrin2 and its interacting proteins were IPed with Sestrin2 antibody or control immunoglobulin (IgG). Input (WCL) and IP complex were analyzed by IB with indicated antibodies against endogenous proteins. Cropped gel images are used in this figure and the gels were run under the same experimental conditions.

To test the necessity of AMPK in Sestrin2-mediated mTORC1 control, we treated *AMPK*-null mouse embryonic fibroblasts (*Ampkα_1_^−/−^/Ampkα_2_^−/−^* MEFs) with Sestrin2-overexpressing adenoviruses (Ad-SESN2). Strikingly, *AMPK*-null MEFs were still capable of downregulating mTORC1-dependent phosphorylation of p70 S6 kinase (S6K) upon Sestrin2 expression as much as WT MEFs (Fig. 1A). Thus, even though Sestrin2 activates AMPK^7^,^9^ and constitutive AMPK activation can inhibit chronic mTORC1^4^,^6^, AMPK is not solely responsible for Sestrin2-induced mTORC1 suppression.

### Identification of GATOR2 components as Sestrin2-binding proteins

To identify new mediators of Sestrin2 function, we conducted a tandem affinity purification (TAP)-mass spectrometry (MS) experiment^18^. In the experiment, there were only six proteins, namely MIOS, WDR24, WDR59, SEH1L, SEC13 and PPM1A, whose unique peptide sequences were represented in the Sestrin2-interacting proteome more than three times (Fig. 1B). Because each of these proteins showed a very weak to hardly detectable physical interaction with Sestrin2 when co-expressed in human embryonic kidney 293 (HEK293) cells (Fig. S1A), we initially judged that the interactions between Sestrin2 and these proteins were insignificant. However, after realizing that five of these proteins form a protein complex named GATOR2^10^, we hypothesized that an intact GATOR2 complex may be required for stable interaction with Sestrin2. Indeed, when all GATOR2 components were co-expressed with Sestrin2 in HEK293 cells, all five proteins were strongly co-immunoprecipitated (IPed) with Sestrin2 (Fig. 1C). This specific interaction was also observed in an *in vitro* pull-down assay (Fig. S1B). Endogenous GATOR2 components were also co-IPed with endogenous Sestrin2 in mouse liver tissues (Fig. 1D) and embryonic fibroblasts (MEF) (Fig. 1E and S2), confirming the existence of endogenous physical association between Sestrin2 and GATOR2.

### GATOR1 does not interact with Sestrin2

Sestrin1, a closely related homolog of Sestrin2 with the same ability to suppress mTORC1^7^, also interacted with GATOR2 complex in HEK293 cells (Fig. S3A). We thought that one of the subdomains in Sestrin1 and Sestrin2, which were identified through a phylogenetic analysis^19^, could be solely responsible for binding to GATOR2 (Fig. S3B). However, truncated Sestrin2 proteins were unable to bind GATOR2, suggesting that only intact full-length Sestrin2 can physically interact with GATOR2 (Fig. S3C). We were also curious if Sestrins can also interact with GATOR1, another GATOR subcomplex loosely bound to GATOR2^10^. However, when co-expressed with Sestrin2 and GATOR2 in HEK293 cells, the three GATOR1 components, DEPDC5, NPRL2 and NPRL3, were not found in the anti-Sestrin2 IP complex that contained all GATOR2 components (Fig. S3D). Consistent with this result, none of the GATOR1 components were represented in the total of ~599 peptides identified from the TAP-MS experiment described above. Therefore, Sestrin2 specifically associates with GATOR2 but not GATOR1.

### Sestrin2 binding to GATOR2 attenuates formation of the GATOR1-GATOR2 complex

**Figure 2:**
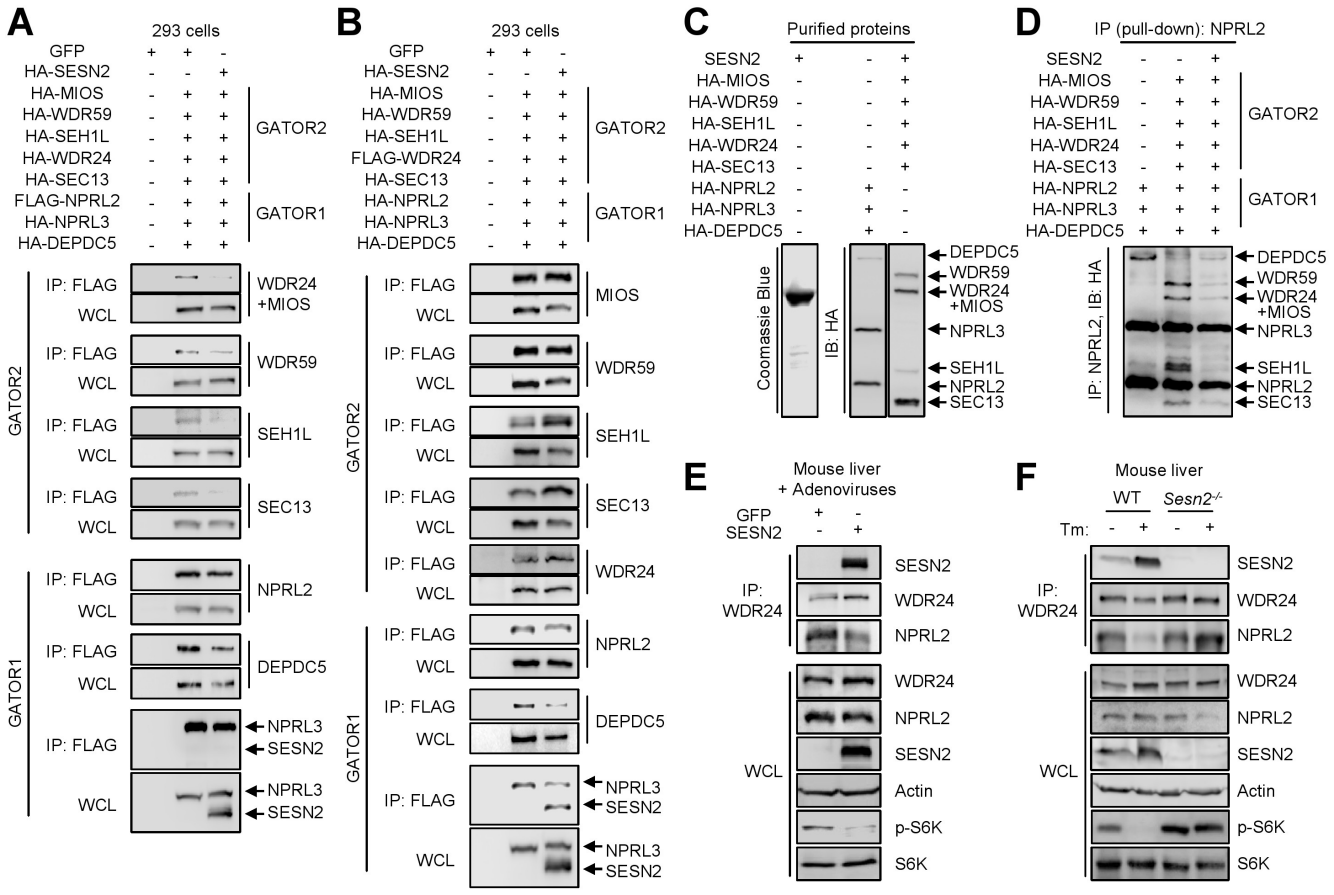
Sestrin2 decreases GATOR1-GATOR2 binding. (A and B) Sestrin2 destabilizes physical interaction between GATOR1 and GATOR2 in HEK293 cells. HA-tagged Sestrin2 was co-transfected with GATOR1 and GATOR2 components as indicated. NPRL2, a GATOR1 component, or WDR24, a GATOR2 component, was IPed using Flag antibody in (A) and (B), respectively. Input (WCL) and IP complex were analyzed by IB. (C) Purification of Sestrin2, GATOR1 and GATOR2 proteins. Sestrin2 and HA-GATOR1/2 complexes were purified from transformed *E. coli* and transfected HEK293 cells, respectively. (D) Sestrin2 inhibits assembly of GATOR1-GATOR2 supercomplex *in vitro*. As indicated, the purified proteins were mixed and incubated together. GATOR1 was pulled down from the mixture using NPRL2 antibody. GATOR1 and co-purified GATOR2 was detected through IB with HA antibody. (E) Sestrin2 overexpression decreases GATOR1-GATOR2 association in mouse liver. 10^9^ pfu of adenoviruses expressing GFP or Sestrin2 were injected into two-month-old WT mice through the tail vein. After 4 days, liver lysates were prepared, and endogenous GATOR2 was IPed with WDR24 antibody. WCL and IP complex were analyzed by IB. (F) Sestrin2 is required for the effect of tunicamycin (Tm) on GATOR1-GATOR2 interaction. Two-month-old WT or *Sesn2^−/−^* mice were injected with Tm (500 mg/Kg i.p.) as described^6^. After 24 hrs, liver lysates were prepared, and GATOR2 was IPed with WDR24 antibody. WCL and IP complex were analyzed by IB. Cropped gel images are used in this figure and the gels were run under the same experimental conditions.

Because the Sestrin2-GATOR2 complex does not show any evidence of physical association with GATOR1, it is plausible that Sestrin2 can inhibit the binding of GATOR1 to GATOR2. Indeed, when co-transfected with GATOR1 and GATOR2 in HEK293 cells, Sestrin2 diminished the association between GATOR1 and GATOR2 (Fig. 2A, 2B) in a dose-dependent manner (Fig. S4). Sestrin2 itself was found in the anti-GATOR2 IP complex (Fig. 2B and S4) but not in the anti-GATOR1 IP complex (Fig. 2A). *In vitro* addition of Sestrin2 protein to immunopurified GATOR1 and GATOR2 protein components (Fig. 2C) also reduced the amount of GATOR2 components pulled-down with NPRL2 (GATOR1) antibodies (Fig. 2D). Sestrin2 overexpression also attenuated formation of endogenous GATOR1-GATOR2 supercomplex; although there is a substantial association between NPRL2 and WDR24 in normal liver, adenovirus-mediated overexpression of Sestrin2 in mouse liver reduced the amount of NPRL2 co-IPed with WDR24 from liver lysates (Fig. 2E). We have recently shown that tunicamycin (Tm)-induced ER stress inhibits mTORC1 by inducing endogenous Sestrin2 (Fig. 2F)^6^. Thus, we tested whether ER stress could modulate GATOR signaling through Sestrin2. In WT mice, Tm-induced Sestrin2 showed strong association with WDR24 (Fig. 2F), and less NPRL2 was bound to WDR24 upon Tm treatment (Fig. 2F). However, Tm-induced decrease in NPRL2-WDR24 association was not observed in *Sesn2^−/−^* mice (Fig. 2F), indicating that GATOR1/2 complexes are indeed a target of endogenous Sestrin2 during hepatic ER stress. These results collectively suggest that increased expression of Sestrin2 can promote release of GATOR1 from the GATOR1-GATOR2 complex by competing with GATOR1 for the association with GATOR2.

### Sestrin2 potentiates GATOR1's GAP activity towards RagB

**Figure 3:**
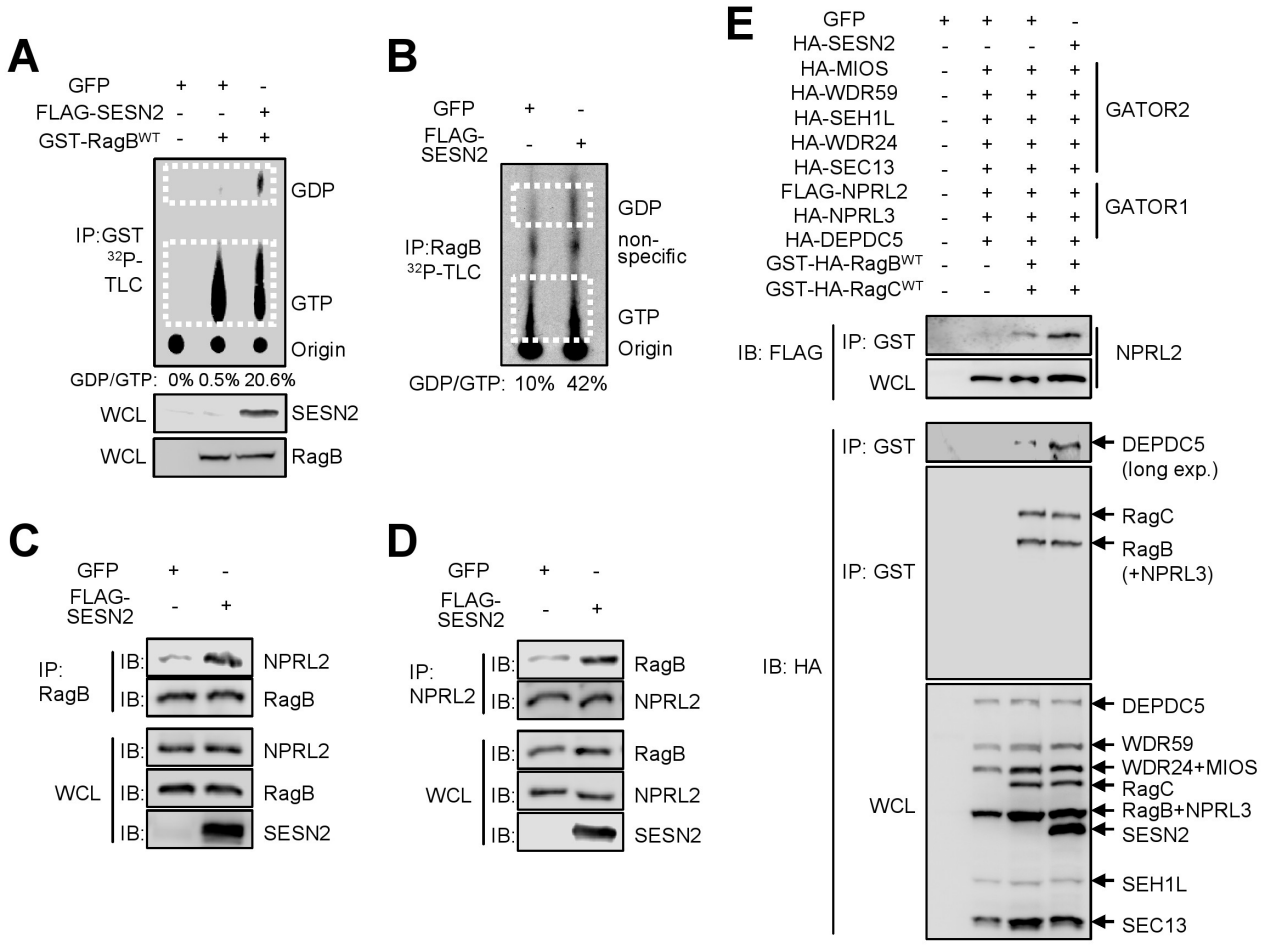
Sestrin2 liberates GATOR1 from GATOR2-mediated inhibition. (A) Inactivation of GST-RagB by Sestrin2. Flag-tagged Sestrin2 was co-transfected with GST-tagged RagB. Following metabolic labeling of the guanine nucleotide pools with ^32^P, GST-RagB was pulled-down using Glutathione-Sepharose 4B beads. RagB-bound nucleotides were separated by thin layer chromatography (TLC) and visualized through autoradiography. The percentage of GDP-bound RagB protein is shown below each lane. Expression of Sestrin2 and GST-RagB were analyzed by IB of WCL. (B-D) Inactivation of endogenous RagB by Sestrin2-potentiated GATOR1. HEK293 cells were transduced with GFP (-) or Sestrin2-overexpressing (+) lentiviruses. After 48 h, RagB was IPed from cell lysates using RagB antibody (B,C) or NPRL2 antibody (D). ^32^P-labeled nucleotides in the IP complex were separated by TLC and visualized through autoradiography (B). Input (WCL) and IP complex were analyzed by IB of endogenous proteins (C,D). (E) Sestrin2 promotes physical association between GATOR1 and RagB/RagC heterodimer. Sestrin2 was co-transfected with GATOR1, GATOR2 and GST-tagged RagB/RagC proteins as indicated. RagB/RagC proteins were pulled-down using Glutathione-Sepharose 4B beads. Input (WCL) and GST-purified complex (IP: GST) were analyzed by IB. Cropped gel images are used in this figure and the gels were run under the same experimental conditions.

As GATOR2 is an inhibitor of GATOR1, Sestrin2-mediated release of GATOR1 from the GATOR1-GATOR2 complex can direct the GAP activity of GATOR1 towards its target RagB. To test this possibility, we tested if Sestrin2 can regulate RagB in cells. Although transfected GST-tagged RagB is predominantly in a GTP-bound state under normal culture conditions, Sestrin2 co-expression significantly elevated the level of RagB:GDP while decreasing the level of RagB:GTP (Fig. 3A). Sestrin2 also promoted hydrolysis of GTP bound to endogenous RagB protein (Fig. 3B), suggesting that Sestrin2 indeed controls RagB. Interestingly, the amount of NPRL2, a GATOR1 component, co-IPed with RagB was substantially increased when Sestrin2 was expressed (Fig. 3C), and the amount of RagB co-IPed with NPRL2 was also increased upon Sestrin2 expression (Fig. 3D), suggesting that Sestrin2 enhanced the association between GATOR1 and RagB. When co-transfected with GATOR1, GATOR2 and GST-tagged RagB/C in HEK293 cells, Sestrin2 increased the amount of NPRL2 and DEPDC5 proteins pulled down with GST-tagged RagB/C proteins (Fig. 3E). These data collectively indicate that Sestrin2 regulates RagB activity by directing the GAP activity of GATOR1 towards RagB.

### Sestrin2 regulates subcellular mTOR localization

**Figure 4:**
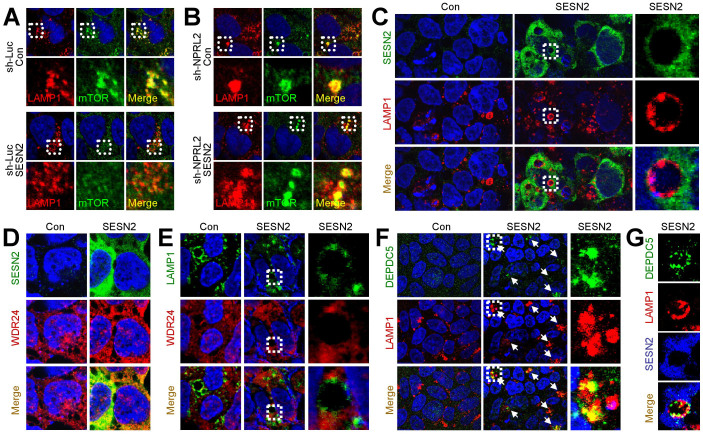
Sestrin2 controls lysosomal localization of mTOR and GATOR1. (A and B) Sestrin2 expels mTORC1 from lysosomal surface through GATOR. HEK293 cells stably transduced with shRNA targeting luciferase (A) or NPRL2 (B) were infected with control or Sestrin2-overexpressing lentiviruses. After 48 h, cells were immunostained with mTOR (green) and LAMP1 (red, a lysosomal marker) antibodies and DAPI (blue). Boxed areas in upper panels are magnified in lower panels. (C) Sestrin2 is excluded from lysosomal membrane. HEK293 cells transduced with control or Sestrin2-overexpressing lentiviruses were immunostained with Sestrin2 (green) and LAMP1 (red) antibodies and DAPI (blue). Boxed areas are magnified in right-most panels. (D and E) Endogenous WDR24 shows substantial co-localization with Sestrin2 but not with lysosomes. HEK293 cells transduced with control or Sestrin2-overexpressing lentiviruses were immunostained with Sestrin2 (D, green) or LAMP1 (E, green; boxed areas are magnified in right-most panels) and WDR24 (red) antibodies and DAPI (blue). (F and G) Endogenous DEPDC5 is accumulated on lysosomal membrane upon Sestrin2 induction. HEK293 cells transduced with control or Sestrin2-overexpressing lentiviruses were immunostained with DEPDC5 (green) and LAMP1 (red) antibodies and DAPI (blue) (F; boxed areas are magnified in right-most panels) or DEPDC5 (green), LAMP1 (red) and Sestrin2 (blue) antibodies (G).

Rag GTPases regulate mTORC1 partially by regulating its subcellular localization. In the presence of amino acids, RagB:GTP recruits mTORC1 to the lysosomal surface where mTORC1 can be activated by RagB, as well as by other GTPases such as Rheb^20^. It is thought that RagB is activated in the presence of sufficient amino acids; however, from the experiments described above, we found that Sestrin2 can inhibit RagB even in the presence of sufficient amino acids. Consequently, Sestrin2 expression expelled mTOR from the lysosomal surface (Fig. 4A). To determine whether GATOR complex regulation is critical for the Sestrin2's role in regulating mTOR localization, we silenced NPRL2, an essential subunit of GATOR1 required for its GAP activity^10^. Sestrin2 was unable to control mTOR localization in NPRL2-silenced condition; NPRL2-silenced cells showed constitutive lysosomal localization of mTOR regardless of Sestrin2 expression (Fig. 4B). These results suggest that GATOR complexes are essential for Sestrin2-mediated control of subcellular mTOR localization.

### Sestrin2 is not present on the lysosomal membrane

Rag GTPases were shown to be present on the lysosomal membrane^21^,^22^. Thus, we were interested in subcellular localization of Sestrin2. Interestingly, Sestrin2 was almost completely excluded from lysosomal membrane (Fig. 4C). Consistent with the strong physical association between Sestrin2 and GATOR2 (Fig. 1), WDR24, a GATOR2 component, showed substantial co-localization with Sestrin2 (Fig. 4D), while also excluded from lysosomal membrane (Fig. 4E). Because GATOR1 was reported to be present on the lysosomal membrane when overexpressed^10^,^14^, and because Sestrin2 indirectly regulates the association between GATOR1 and RagB, it is possible that Sestrin2 changes the subcellular localization of GATOR1 through displacement of GATOR2. Indeed, although endogenous DEPDC5, a GATOR1 component, does not show significant lysosomal localization under normal culture conditions, a substantial amount of DEPDC5 accumulated on the lysosomal membrane when Sestrin2 was overexpressed (Fig. 4F, arrows). Overexpressed Sestrin2 itself was not co-localized with lysosomally-accumulated DEPDC5 (Fig. 4G). These results collectively indicate that Sestrin2 indirectly regulates RagB-mTORC1 signaling by modulating GATOR complexes.

### Sestrin2 regulates mTORC1 activity through GATOR regulation

**Figure 5:**
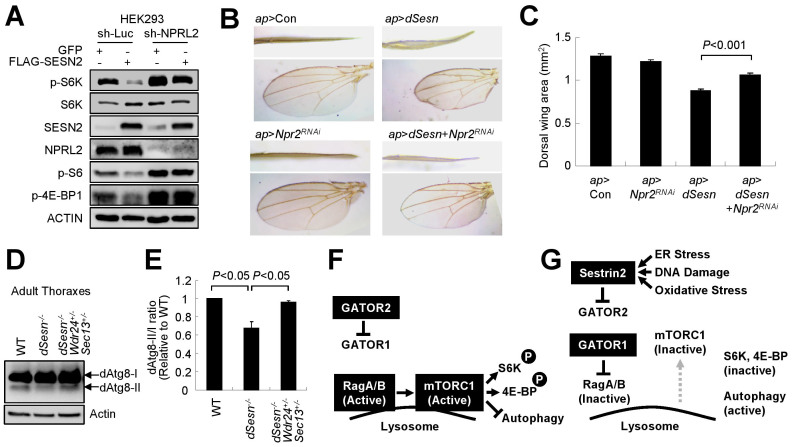
Sestrin2 controls mTORC1 through GATOR complexes. (A) Sestrin2 inhibits phosphorylation of mTORC1 targets through GATOR. HEK293 cells stably transduced with indicated shRNA were infected with GFP or Sestrin2-overexpressing lentiviruses. After 48 h, cells were analyzed by IB with indicated antibodies against endogenous proteins. (B and C) Silencing Npr2, the *Drosophila* homologue of NPRL2, relieves *Drosophila* Sestrin (dSesn)-induced growth arrest. Anterior (B, upper panels) and dorsal (B, lower panels) views of wing blades with *ap-GAL4*–driven expression of indicated transgenic elements were imaged under a dissection microscope and a transmitted light microscope, respectively. Dorsal wing area were quantified and presented as a bar graph (C, n = 7, means ± s.e.m.). *P* value was calculated using Student's t-test. (D and E) Autophagy defects in *dSesn*-null animals were corrected by heterozygotic mutations of GATOR2 components. Thoraces from 5 day-old flies of indicated strains were analyzed through immunoblotting of dAtg8 and Actin proteins (D). Relative levels of unprocessed (dAtg8-I) and processed (dAtg8-II) dAtg8 proteins were quantified by densitometry and presented as a bar graph (E, n = 3, means ± s.e.m.). *P* values were calculated using Student's t-test. (F and G) Proposed model of the Sestrin2-GATOR-RagB pathway that mediates stress-induced mTORC1 silencing. In unstressed conditions, GATOR1 is constitutively inhibited by GATOR2, and RagB recruits mTORC1 to lysosomal surface and activates it as well as its downstream targets (F). Upon stress-induced expression of Sestrin2, GATOR1 is released from GATOR2-mediated inhibition and inactivates RagB (G). In the absence of active RagB, mTORC1 is released from lysosomal surface and mTORC1 signaling is subsequently inactivated. Cropped gel images are used in this figure and the gels were run under the same experimental conditions.

Then we determined whether GATOR regulation is essential for Sestrin2-mediated inhibition of mTORC1 activity. As previously reported^7^, Sestrin2 expression in HEK293 cells suppressed phosphorylation of mTORC1 downstream targets including p70 ribosomal protein S6 kinase (S6K) and eukaryotic translation initiation factor 4E-binding protein (4E-BP) (Fig. 5A). The phosphorylation of ribosomal protein S6, which is mediated by S6K, was also downregulated by Sestrin2 expression (Fig. 5A). RNAi-mediated silencing of NPRL2, which completely abrogates GATOR1 activity^10^, nullified Sestrin2's effect on mTORC1-dependent phosphorylation events (Fig. 5A), supporting the notion that GATOR complexes mediate Sestrin2-induced suppression of mTORC1.

### *Drosophila* Sestrin regulates cell growth through GATOR regulation

Sestrin-mediated inhibition of mTORC1 activity is conserved in *Drosophila* and overexpression of mammalian Sestrin results in inhibition of dTORC1^5^. Because components of GATOR1/2 complexes are also well conserved in *Drosophila*^10^,^14^, we investigated whether GATOR regulation is also important for *Drosophila* Sestrin (dSesn)-mediated cell growth control in *Drosophila*. Especially, we focused on *Drosophila* wing epithelium where dSesn- and dRagB-mediated control of dTORC1 and cell growth has been well characterized^5^,^23^. We utilized an *apterous* (*ap*)-*Gal4* system to specifically modulate gene expression in dorsal epithelium of wing tissue. In this system, enhanced cell growth results in a bent-down wing phenotype, whereas reduced cell growth results in bent-up wing phenotype^5^. *ap-Gal4*-mediated silencing of Npr2/CG9104, a homologue of mammalian NPRL2 that is essential for *Drosophila* GATOR1 activity^10^,^14^, did not alter cell growth of dorsal wing epithelium (Fig. 5B, 5C), suggesting that GATOR1 is constitutively suppressed by GATOR2 during normal development. As formerly observed^5^, *ap-Gal4*-mediated expression of dSesn strongly reduced growth of dorsal wing epithelium, resulting in bent-up wing phenotype and reduced dorsal wing area (Fig. 5B, 5C). dSesn-induced cell growth suppression was significantly ameliorated by silencing of Npr2 (Fig. 5B, 5C), suggesting that dSesn suppresses cell growth through GATOR1. This finding also indicates that the mechanism of GATOR-mTORC1 regulation by Sestrin-family proteins is highly conserved between mammals and *Drosophila*.

### GATOR2 downregulation can correct autophagy defects of *dSesn*-null flies

Because dSesn-mediated suppression of chronic dTORC1 activation is important for proper autophagy, *dSesn*-null mutant flies exhibited an autophagy defect and its associated degenerative phenotypes in the skeletal muscle^5^. We questioned if GATOR2 misregulation is physiologically responsible for endogenous *dSesn*'s autophagy-controlling function. Interestingly, the autophagy downregulation observed in *dSesn*-null skeletal muscle was nearly completely suppressed by the hypomorphic mutation of GATOR2 components (transheterozygotic mutation of *Wdr24* and *Sec13*), as manifested by restoration of the dAtg8-II/I ratio (Fig. 5D, 5E). This genetic interaction result suggests that one of Sestrin's main physiological functions is the suppression of GATOR2.

## Discussion

Sestrins link environmental stresses to inhibition of mTORC1. For instance, Sestrin2 suppresses mTORC1 activity in response to DNA damage^7^,^9^, ER stress^6^,^24^, nutritional stress^4^,^25^ or energetic stress^26^. Sestrin-mediated suppression of mTORC1 activity is important for maintaining cellular and organismal homeostasis during stress. Loss of Sestrin2 in mice or its homologue in *Drosophila* causes chronic upregulation of mTORC1/dTORC1 that results in diverse age- and obesity-associated pathologies^4^,^5^,^6^,^25^. The current study provides a molecular mechanism through which Sestrins inhibit mTORC1 activity. Under normal condition, GATOR1 and GATOR2 form a GATOR supercomplex in which GATOR1's GAP activity is inhibited by GATOR2 (Fig. 5F). With GATOR1's activity suppressed, RagB is activated by its guanylate exchange factor (GEF) Ragulator and can recruit mTORC1 to the lysosomal surface^22^. On the lysosomal surface, mTORC1 is activated by its association with RagB and Rheb and can phosphorylate downstream targets such as S6K and 4E-BP. However, in response to environmental stresses, Sestrin2 is induced and associates with GATOR2 to form a Sestrin2-GATOR2 complex, thereby freeing GATOR1 to become an active GAP that directly binds to RagB and enhances its GTPase activity (Fig. 5G). GTP hydrolysis results in inactivation of RagB (RagB:GDP), releasing mTORC1 from the lysosomal surface and causing mTORC1 inactivation. Sestrin2-dependent AMPK activation^7^,^9^ provides an additional mechanism for mTORC1 suppression through regulation of TSC2-Rheb^27^ and Raptor^28^. Although AMPK is dispensable for Sestrin2-mediated mTORC1 inhibition in MEF cells, the AMPK-dependent mechanism could at least partially contribute to the mTORC1 regulation in certain physiological contexts, such as during DNA damage^7^,^9^.

RagB GTPase signaling provides a molecular conduit that allows amino acids to regulate mTORC1. However, other types of regulatory inputs, such as growth factors and environmental stresses also modulate mTORC1 signaling and are thought to act through a TSC2-Rheb module rather than RagB^20^. Several regulators of RagB have been identified, such as Ragulator^22^, GATOR1/2 complexes^10^ and Folliculin^29^. Although Ragulator and Folliculin complexes were shown to mediate amino acid-mediated control of RagB-mTORC1, how GATOR complexes are physiologically regulated has been obscure. Thus, this study demonstrates that RagB is involved in stress-dependent mTORC1 regulation and shows that GATOR activity itself can be regulated.

While this work was under review, three other papers presented findings that Sestrin2 can control mTORC1 signaling through regulation of RagB^30^,^31^,^32^. One paper proposed that Sestrin2 is a direct guanine nucleotide dissociation inhibitor (GDI) for RagB^32^. Some results of our current paper, such as that Sestrin2 regulates mTORC1 localization (Fig. 4A) and that GATOR1 is required for Sestrin2's mTORC1-inhibiting function (Fig. 4B and 5A-5C; absence of GAP may nullify the effect of GDI), could be alternatively explained by Sestrin2 possessing GDI activity. However, none of the Rag GTPases, including RagB, were identified from in our TAP-MS purification of Sestrin2-interacting proteins, suggesting that if there is an interaction between Sestrin2 and RagB, it would be weaker than the interaction between Sestrin2 and endogenous GATOR2. Our pull down experiment also indicated that Sestrin2 does not stoichiometrically form a stable complex with RagB/C GTPases in cells (Fig. 3E). However, our current findings do not completely rule out the possibility that the proposed GDI activity of Sestrin2 also contributes to the RagB-mTORC1 regulation.

The other two papers, in consistence with our findings, suggest that GATOR2 is the molecular target of Sestrin2^30^,^31^. However, one important point of contention was that they were unable to observe that Sestrin2 modulates the affinity between overexpressed GATOR1 and GATOR2 proteins. One of the possible reasons for this discrepancy is that Sestrin2 cannot expel GATOR1 from the protein complex when expressed at relatively low levels compared to the overexpressed amounts of GATOR proteins. Indeed, Sestrin2-mediated dissociation between GATOR1 and GATOR2 was found to be moderate when the level of Sestrin2 expression was comparable to the level of GATOR1/2 expression (Fig. 2B and S4). As Sestrin2 is known to inhibit mTORC1 signaling when robustly expressed upon stresses, it is plausible that the GATOR1/2-dissociating activity of Sestrin2 is only observed when its amount far exceeds the level of GATOR1/2. Importantly, we have shown that, when either stress-induced or ectopically-expressed, Sestrin2 can strongly diminish the binding between endogenous GATOR1 and GATOR2 components (Fig. 2E, 2F).

Although our results indicate that the level of Sestrin2 expression is important for proper GATOR1/2 modulation and mTORC1 inhibition, it is possible that the molecular relationship between Sestrin2 and GATOR2 is affected by factors other than the stoichiometric Sestrin2 levels. The aforementioned recent papers showed that Sestrin2 can mediate mTORC1 inhibition upon amino acid starvation, a stress that does not induce transcriptional Sestrin2 upregulation^30^,^31^,^32^, and that the association between Sestrin2 and GATOR2 is enhanced upon amino acid starvation^30^. It is certainly possible that Sestrin2 is subjected to a post-translational regulation that regulates its GATOR-modulating activity. For example, it has been recently shown that Sestrin2 can be phosphorylated by ULK1, a protein kinase that is activated upon starvation^33^.

## Methods

### Antibodies and Reagents

Sestrin2 antibodies were formerly described^6^. AMPKα (sc-25792), LAMP1 (sc-20011), S6K (sc-230), WDR24 (sc-244614), WDR59 (sc-137927), NPRL2 (sc-376986), SEH1L (sc-79055) and DEPDC5 (sc-86115) antibodies were obtained from Santa Cruz Biotechnology, Inc. mTOR (2983), phospho-S6K (9234), MIOS (13557), RagB (8150), phospho-S6 (2211) and phospho-4E-BP1 (2855) antibodies were purchased from Cell Signaling Technology, Inc. Actin (JLA20) and Myc (9E10) antibodies were purchased from Developmental Studies Hybridoma Bank (DSHB). Hemagglutinin (HA) antibody (3F10) was from Roche. Flag (M2) antibody, HA (A2095) and Flag (A2220) antibody-conjugated beads and Tm were purchased from Sigma. Anti-GABARAP/dAtg8a antibody (ab109364) was from Abcam. Adenoviruses expressing human Sestrin2 (130233A) were purchased from Applied Biological Materials Inc. Epitope-tagged RagB/C and GATOR1/2 plasmids (19301, 19304, 46327-46333 and 46335; all in the pRK5 expression vector), which were originally generated in Dr. Sabatini's lab^10^, were purchased from Addgene.

### Lentiviruses

The lentiviral construct for sh-NPRL2 (Broad Institute TRC Portal accession code TRCN0000234677) was purchased from Open Biosystems (Huntsville, AL). Lentiviral constructs expressing Flag-tagged Sestrin1 and Sestrin2 were formerly described^4^. Lentiviruses were generated and amplified in the Vector Core facility at the University of Michigan (UM).

### Adenoviruses

shRNA adenoviruses were constructed using the BLOCK-iT Adenoviral RNAi Expression System (K4941-00, Invitrogen) according to the manufacturer's instructions. Target sequences against mouse genes including *Wdr24* (GCACCAGATGGATGAGAATCT), *Mios* (GGGTTCACCTTTAGATGTTCT), *Depdc5* (GCAGCGGATGATTGATAATGG) and *Nprl2* (GCAGATCCTGCCCTACATTGA) were designed using the BLOCK-iT™ RNAi Designer (Invitrogen).

### Cell culture and Transfection

HEK293 and MEF cells were cultured in Dulbecco's modified Eagle's medium (DMEM, Invitrogen) containing 10% fetal bovine serum (FBS) and penicillin/streptomycin at 37°C in 5% CO_2_. *Ampkα_1_^-/−^/Ampkα_2_^-/−^* MEF cells were previously described^4^,^34^. For transient expression of proteins, HEK293 cells were transfected with purified plasmid constructs and polyethylenimine (PEI, Sigma) as previously described^35^. Cells were harvested 24 hrs after transfection for biochemical assays.

### TAP-MS Analysis

TAP-MS analysis was performed according to the method that we have formerly described^18^. In brief, MCF10A cells were stably infected with pBABE-Flag-SBP-Sestrin2 retroviruses. Cells were then cultured to 80% confluency and lysed with buffer containing 0.3% CHAPS. Lysate was first incubated with Flag antibody-conjugated resin and Flag-SBP-Sestrin2 was eluted with elution buffer containing 200 ng/μl 3X-Flag peptide. The eluate was further incubated with streptavidin-conjugated resin and Flag-SBP-Sestrin2 was again eluted with buffer containing 4 mM biotin. The TAP-purified Flag-SBP-Sestrin2 and associated proteins were trypsin digested and analyzed by LC/ESI MS/MS. Proteins identified in both vector control and Flag-SBP-Sestrin2 experiments were determined to be false-positive.

### Immunoprecipitation (IP)

Cell and tissue lysates were prepared in a lysis buffer^5^ containing 0.3% CHAPS and protease inhibitor cocktail (Roche), and IPed with anti-HA (A2095, Sigma) or anti-Flag (A2220, Sigma) agarose bead or other antibodies conjugated to a protein G/A bead (Calbiochem). GST-tagged proteins were pulled down with Glutathione-Sepharose 4B bead (Amersham). The immunocomplexes were then washed four times with the lysis buffer and analyzed through IB^33^.

### Immunoblotting (IB)

Cell or tissue lysates and IP or pull-down complexes were boiled in SDS sample buffer for 5 min, separated by SDS-PAGE, transferred to PVDF membranes and probed with primary antibodies. After incubation with secondary antibodies conjugated with HRP, chemiluminescence was detected using LAS4000 (GE) systems and quantified by densitometry^4^. For co-IP and pull-down experiments, non-specific background binding level was set as signal threshold for the presented immunoblot images.

### Protein Purification and *in vitro* Pull-down Assay

Full-length Sestrin2 cDNA was cloned into a pET vector modified for ligation-independent cloning, which has both an N-terminal His_6_ tag and MBP tag with a tobacco etch virus (TEV) protease cleavage site. Recombinant Sestrin2 was purified from transformed Rosetta (DE3) pLysS *E. coli* (Novagen), using Ni-NTA column and amylase resin. Final eluates were treated with TEV and His_6_-MBP fragments were removed by Ni-NTA column. Target proteins (Sestrin2) were further purified by HiTrap Q column (GE healthcare) and a Superdex 200 size exclusion column (Prep grade 16/60: GE healthcare). 1 μg of Sestrin2 protein was used for all *in vitro* assays including pull-down and GTPase assays. HA-GATOR1 and HA-GATOR2 proteins were immunopurified from transfected HEK293 cells using anti-HA agarose beads (A2095, Sigma) and HA peptide (I2149, Sigma). All purified proteins were snap-frozen and stored at -80°C in buffer A [30 mM Tris-HCl pH8.0, 100 mM NaCl, 1 mM TCEP]. For the pull-down assays, proteins were mixed in 20 μl of buffer A and incubated overnight in the presence of antibodies against the target proteins. Target proteins and their associated partners were pulled down by protein G/A bead (Calbiochem) and analyzed by IB.

### Detection of RagB-bound Nucleotides

Determination of guanyl nucleotide binding state of RagB in cells was performed using the formerly described *in vivo* ^32^P metabolic labeling method^36^. In brief, the cells were serum starved overnight, and in the following morning, cells were incubated in phosphate-free medium for 30 min and then in phosphate-free medium containing 0.2 mCi ^32^P (Perkin Elmer) in 60-mm dish. After labeling, the cells were lysed, and GST-recombinant or endogenous RagB proteins were purified through GST pull down and anti-RagB IP, respectively. RagB-bound nucleotides were released by denaturing the protein in elution buffer (2 mM EDTA, 1 mM GDP, 1 mM GTP, 0.2% SDS, 5 mM DTT) at 65°C for 5 min. Polyethylenimine (PEI) cellulose plates and a chromatography chamber saturated with 0.75 M KH_2_PO_4_ (pH 3.4) were used for the thin layer chromatography (TLC). Nucleotide spots were visualized using autoradiography.

### Immunocytochemistry

HEK293 and MEF cells with indicated modulation were seeded onto glass coverslips. On the following day, cells were washed with PBS and fixed with 4% paraformaldehyde. After permeabilizing the cells with 0.3% Triton X-100, they were incubated overnight with primary antibodies. The cells were then washed with PBS and were incubated with Alexa Fluor-conjugated secondary antibodies (Invitrogen) for 30 min and counterstained with DAPI (Invitrogen)^37^. For detection of endogenous WDR24 in Fig. 4D and 4E, Tyramide Signal Amplification (TSA) system (Perkin Elmer) was used to amplify the fluorescence intensity according to the manufacturer's recommendation. WDR24 staining in Fig. S2E was done using conventional Alexa-488 secondary antibodies (Invitrogen). Samples were analyzed under a FluoView 500 laser confocal microscope (Olympus).

### Drosophila and Mice

*w^1118^*, *ap-GAL4* and *UAS-dSesn^WT^* strains were described^4^,^5^. *UAS-Npr2^RNAi^* was generated by Transgenic RNAi project (TRiP) at Harvard Medical School and obtained from the Bloomington stock center. Null mutant strains of *Wdr24* (*CG7605^f06205^*) and *Sec13* (*Sec13^01031^*) were also from the Bloomington stock center. The flies were reared on standard cornmeal-agar medium with humidity (70%), temperature (25°C) and light (12/12 h light/dark cycle) control. *Sesn2^+/+^*, *Sesn2^−/−^*, *Lep^ob/ob^*/*Sesn2^+/−^* and *Lep^ob/ob^*/*Sesn2^−/−^* mouse strains in a C57BL/6 background were described^4^,^6^. Male mice of indicated age were used for the study. Mice were maintained in filter-topped cages and were given free access to autoclaved regular chow diet and water at the University of Michigan (UM) according to the NIH and institutional guidelines. All animal studies were approved and overseen by the University Committee on Use and Care of Animals at UM.

## Author Contributions

J.S.K. and S.H.R. performed mammalian cell culture experiments and biochemical analyses. S.H.R. performed immunostaining experiments. M.Kim performed Drosophila experiments. H.W.P. performed mouse experiments. I.A.S. and H.P. assisted with immunoblotting experiments. U.S.C. provided the purified Sestrin2 proteins. W.W., K.L.G. and M.Karin provided the TAP-MS data. J.S.K., S.H.R., I.A.S., H.P. and J.H.L. prepared the manuscript. J.H.L. developed and directed the research project. All authors discussed the experimental results and reviewed the manuscript.

## Supplementary Material

Supplementary Information Supplementary Figures and Legends
