# Corrigendum: Sestrin2 inhibits mTORC1 through modulation of GATOR complexes

**DOI:** 10.1038/srep14029

**Published:** 2015-09-21

**Authors:** Jeong Sig Kim, Seung-Hyun Ro, Myungjin Kim, Hwan-Woo Park, Ian A. Semple, Haeli Park, Uhn-Soo Cho, Wei Wang, Kun-Liang Guan, Michael Karin, Jun Hee Lee

Scientific Reports 5: Article number: 950210.1038/srep09502; published online: 03302015; updated: 09212015

The results presented in Fig. 1B were produced by Wei Wang, Kun-Liang Guan and Michael Karin. The authors neglected to indicate that the same results were also reported by Parmigiani *et al.* (reference 31), but in that paper the relevant data are not shown.

