# Supplementary material for: Sestrin2 inhibits mTORC1 through modulation of GATOR complexes
Source: Sci Rep. 2015 Mar 30;5:9502. doi: 10.1038/srep09502 (PMC4377584; doi:10.1038/srep09502)
Supplement: Supplementary Information — Supplementary Figures and Legends [file srep09502-s1.doc]

**Supplemental Information to****:**

Sestrin2 inhibits mTORC1 through modulation of GATOR complexes

Jeong Sig Kim, Seung-Hyun Ro, Myungjin Kim, Hwan-Woo Park, Ian A. Semple, Haeli Park, Uhn-Soo Cho, Wei Wang, Kun-Liang Guan, Michael Karin, Jun Hee Lee.

**Supplemental Figures and Legends**

**
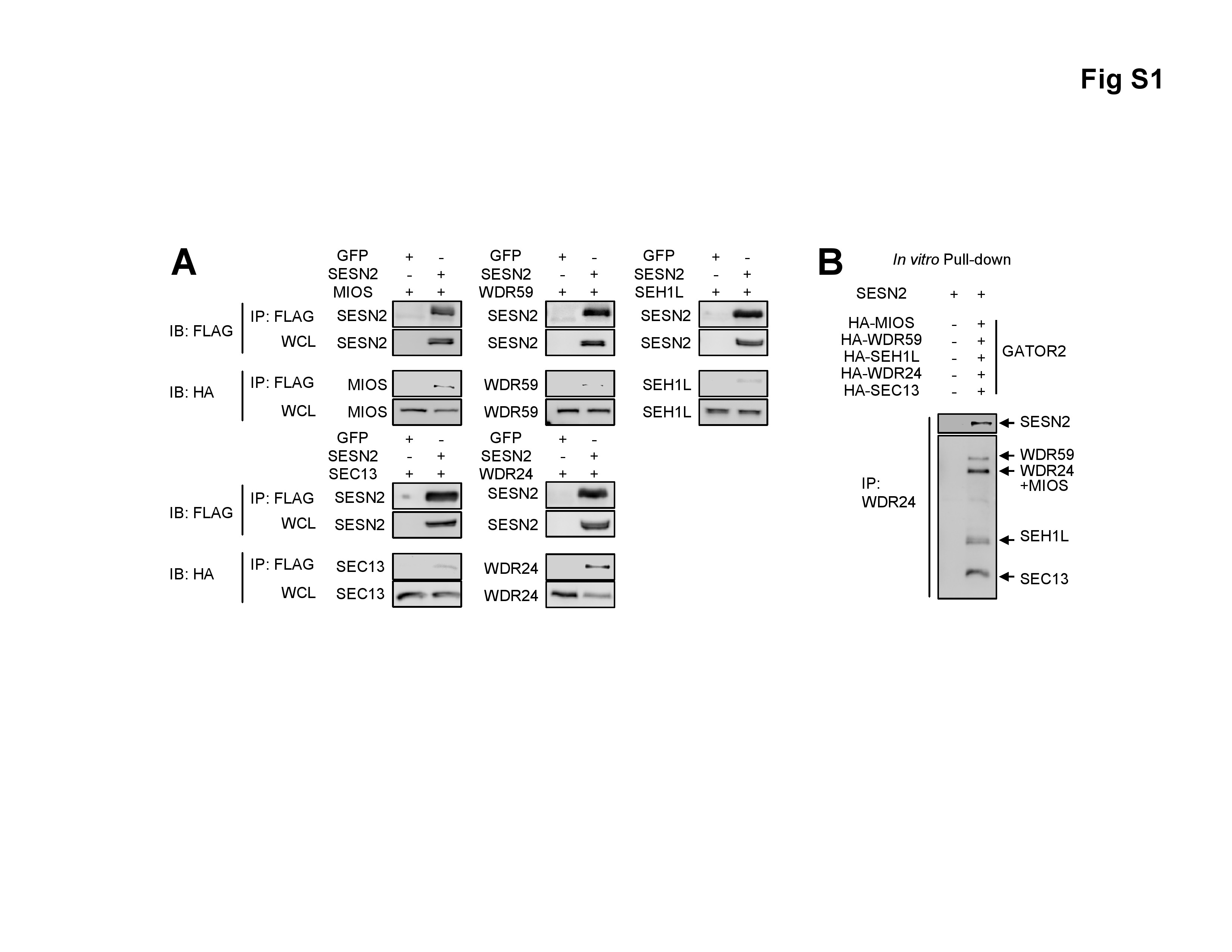
**

**Figure S1 | Interaction between Sestrin2 and GATOR2 components. |** (**A**) Individual GATOR2 components show a very weak physical association with Sestrin2 in HEK293 cells. Flag-tagged Sestrin2 was co-transfected with HA-tagged individual GATOR2 components as indicated. Sestrin2 was immunoprecipitated (IPed) with Flag antibody. Input (whole cell lysates; WCL) and IP complex were analyzed by IB. (**B**) Sestrin2 interacts with GATOR2 *in vitro*. Purified Sestrin2 and GATOR2 proteins (see Fig. 2C for purification data) were incubated together. GATOR2 was pulled down from the mixture using WDR24 antibody. GATOR2 and co-purified Sestrin2 were detected through IB with HA and Sestrin2 antibodies, respectively. Cropped gel images are used in this figure and the gels were run under the same experimental conditions.

**
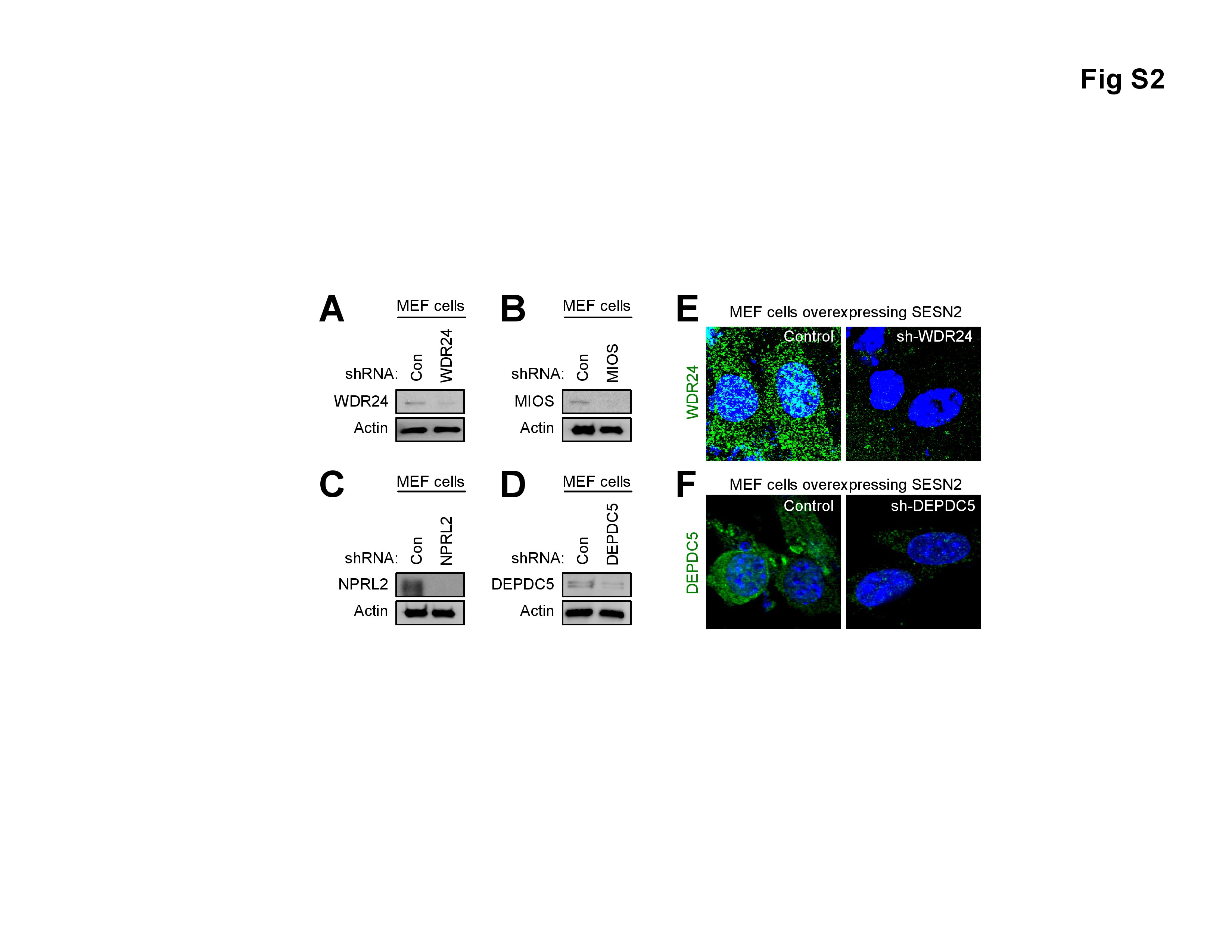
**

**Figure S2 | Antibody tools for detecting endogenous GATOR1/2 components. |** (**A-D**) WT MEF cells were infected with adenoviruses expressing shRNAs targeting human lamin (Con) or mouse GATOR1/2 components including WDR24, MIOS, NPRL2 and DEPDC5. After 48 hrs, cell lysates were analyzed by immunoblotting against endogenous proteins. (**E** and **F**) WT MEF cells were infected with adenoviruses expressing control or GATOR1/2-targetting shRNAs. After 48 hrs, cells were again infected with lentiviruses overexpressing Sestrin2. Then, after 48 hrs, cells were subjected to immunostaining against endogenous proteins. See Materials and Methods for details of adenoviral constructs, antibodies and immunoblotting/immunostaining conditions. Cropped gel images are used in this figure and the gels were run under the same experimental conditions.


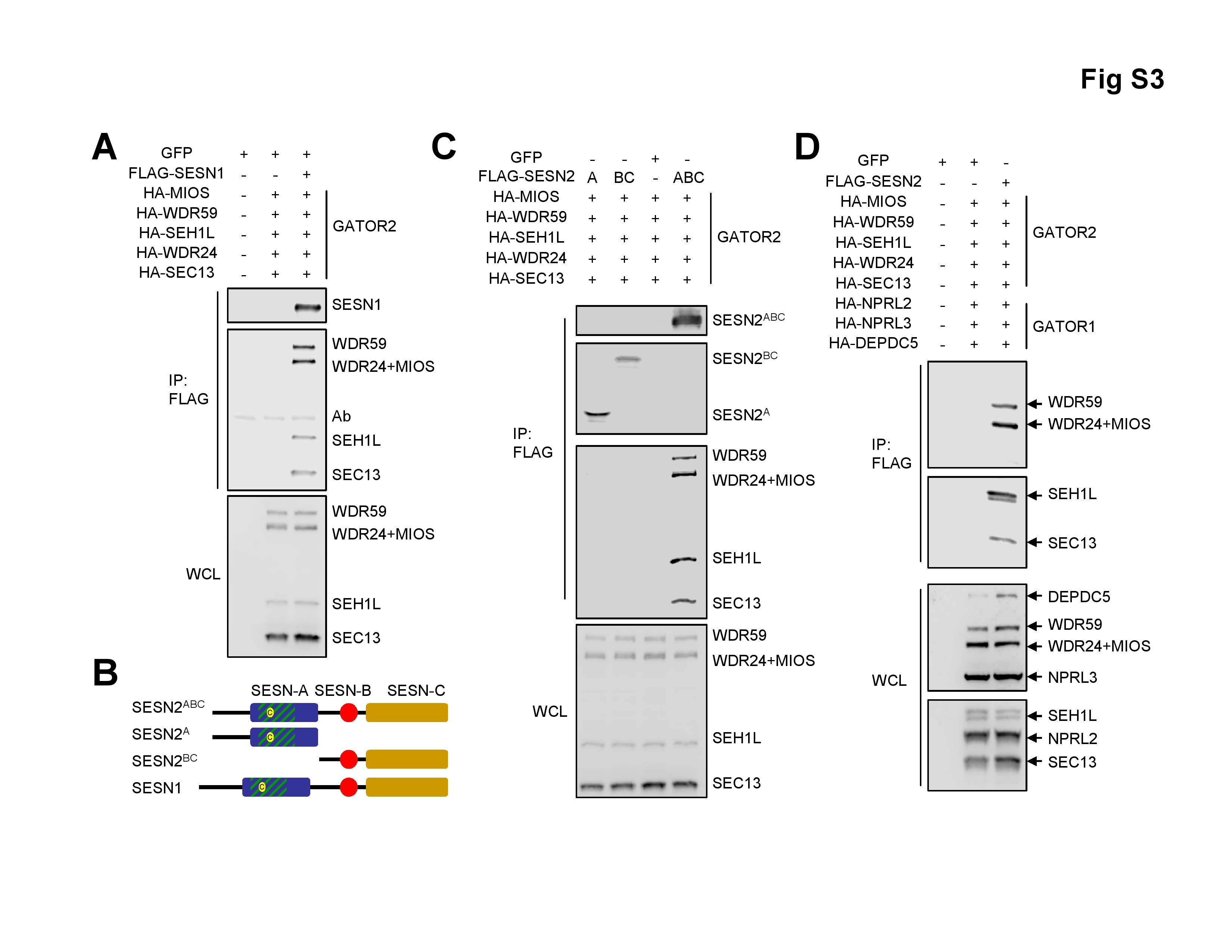


**Figure S3** | **Sestrin1 interacts with GATOR2, but GATOR1 does not interact with Sestrin2.** (**A**) GATOR2 shows strong physical association with Sestrin1 in HEK293 cells. Sestrin1 was co-transfected with GATOR2 components as indicated. Sestrin1 was IPed using Flag antibody. Input (WCL) and IP complex were analyzed by IB. (**B**) Diagram depicting domain structure of wild type and truncated mutants of Sestrins, which was formerly determined through phylogenic analyses^19^. (**C**) Truncated Sestrin2 does not interact with GATOR2 in HEK293 cells. Full-length (ABC) and truncated (A and BC) Sestrin2 was co-transfected with GATOR2 components as indicated. Sestrin2 was IPed using Flag antibody. Input (WCL) and IP complex were analyzed by IB. (**D**) GATOR1 does not interact with Sestrin2 in HEK293 cells. Sestrin2 was co-transfected with GATOR1 and GATOR2 components as indicated. Sestrin2 was IPed using Flag antibody. Input (WCL) and IP complex were analyzed by IB. Cropped gel images are used in this figure and the gels were run under the same experimental conditions.


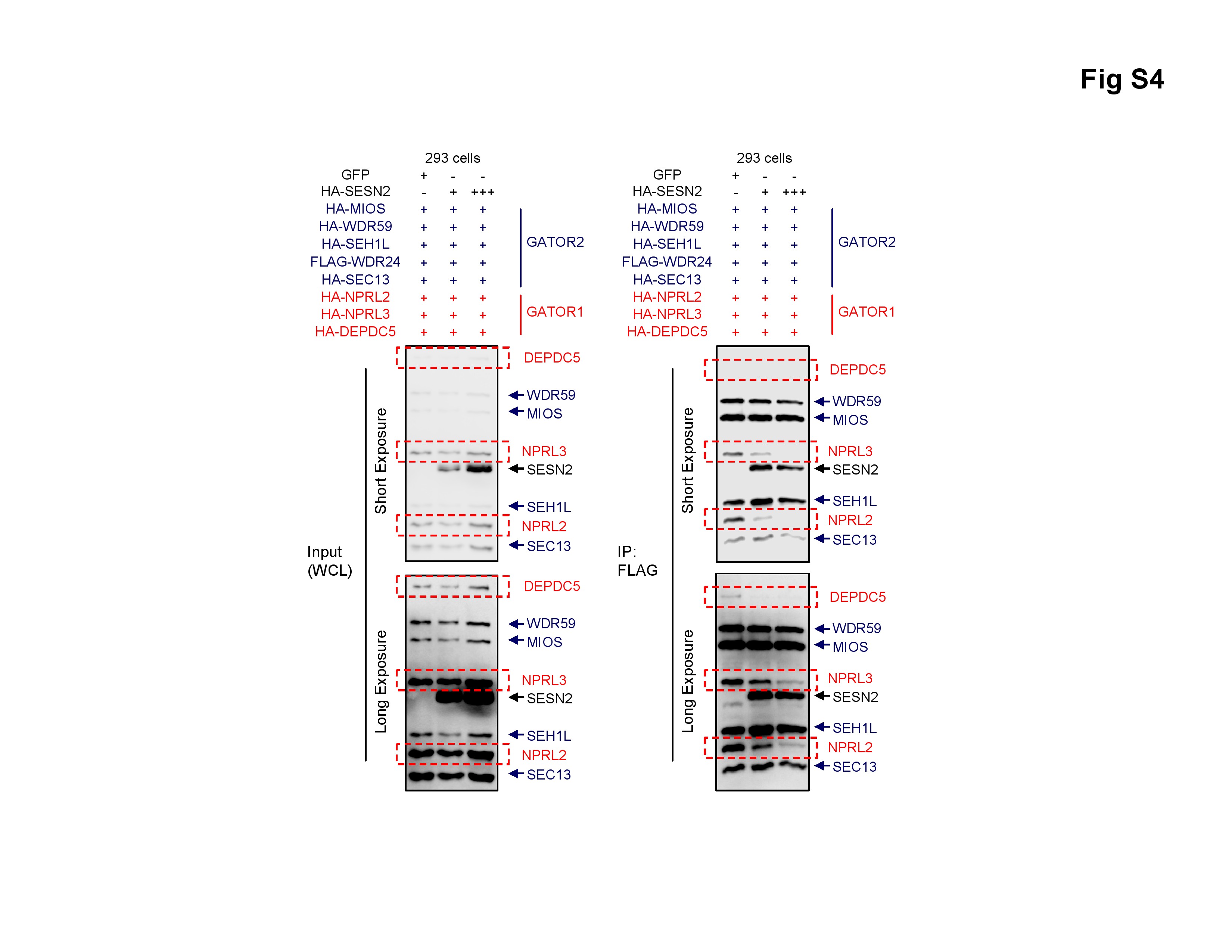


**Figure S4 | Sestrin2 overexpression induces dissociation between GATOR1 and GATOR2.** Sestrin2 destabilizes the GATOR1-GATOR2 supercomplex in HEK293 cells. Through transient transfection, different amounts of HA-tagged Sestrin2 were co-expressed with GATOR1 and GATOR2 components as indicated. WDR24, a GATOR2 component, was IPed using Flag antibody. Input (WCL) and IP complex were analyzed by IB. Protein loading was normalized to the amount of GATOR2 proteins. Short and long exposures of the same blots were shown.
